# Beta Agonist Lung Injury TrIal-2 (BALTI-2) trial protocol: A randomised, double-blind, placebo-controlled of intravenous infusion of salbutamol in the acute respiratory distress syndrome

**DOI:** 10.1186/1745-6215-12-113

**Published:** 2011-05-09

**Authors:** Gavin D Perkins, Simon Gates, Sarah E Lamb, Chris McCabe, Duncan Young, Fang Gao

**Affiliations:** 1 University of Warwick, Warwick Medical School Clinical Trials Unit, Warwick, CV4 7AL, UK; 2 Heart of England NHS Foundation Trust, Bordesley Green East, Birmingham, B9 5SS, UK; 3 Leeds Institute of Health Sciences, University of Leeds, Leeds LS2 9LT, UK; 4 Oxford John Radcliffe Hospital, Oxford, UK

## Abstract

**Background:**

The Acute Respiratory Distress Syndrome (ARDS) is a common cause of respiratory failure in critically ill patients. Experimental studies suggest that treatment with beta agonists may be helpful in ARDS. The Beta Agonist Lung Injury TrIal (BALTI-2) is a multicentre, pragmatic, randomised, double-blind, placebo-controlled clinical trial which aims to determine if sustained treatment with intravenous (IV) salbutamol will improve survival in ARDS.

**Methods/Design:**

Patients fulfilling the American-European Consensus Conference Definition of ARDS will be randomised in a 1:1 ratio to receive an IV infusion either of salbutamol (15 μg kg ideal body weight^-1 ^hr^-1^) or placebo (0.9% sodium chloride solution), for a maximum of seven days. Allocation to randomised groups will use minimisation to ensure balance with respect to hospital of recruitment, age group (<64, 65-84, >85 years) and PaO_2_/FiO_2 _ratio (≤6.7, 6.8- 13.2, ≥13.3 kPa). Data will be recorded by participating ICUs until hospital discharge, and all surviving patients will be followed up by post at six and twelve months post randomisation. The primary outcome is mortality at 28 days after randomisation; secondary outcomes are mortality in ICU, mortality in hospital, number of ventilator-free days, number of organ failure-free days, mortality at twelve months post-randomisation, quality of life at six and twelve months, length of stay in ICU, length of stay in hospital, adverse effects (tachycardia, arrhythmia or other side effects sufficient to stop treatment drug). 1,334 patients will be recruited from about fifty ICUs in the UK. An economic evaluation will be conducted alongside the trial.

**Trial Registration:**

Current Controlled Trials ISRCTN38366450.

## Background

The acute respiratory distress syndrome (ARDS) is a condition characterised by a failure of pulmonary oxygen exchange due to increased alveolar-capillary permeability and resultant lung oedema [1]. It can be caused by primary lung conditions such as aspiration, pneumonia, or can arise as a complication of non-pulmonary conditions such as severe sepsis. ARDS is defined by the 1994 American-European Consensus Conference [2] as the acute onset of hypoxaemia (PaO_2_:FiO_2 _ratio of < 200mm Hg), bilateral infiltrates on a chest radiograph in the absence of cardiogenic causes of pulmonary oedema.

ARDS is common, The ALIVE study reported 13.3% of patients who require mechanical ventilation have ARDS [3]. Intensive Care Unit (ICU) mortality is estimated at 41-46%, corresponding to about 2,200 deaths per year in the UK [4,5]. Patients with ARDS consume significantly more resources than matched patients without ARDS since they require a longer ICU and hospital stay (median 17 vs 8 days and 31 vs 25 days, respectively) [3], and convalescence on the ward and subsequent rehabilitation in the community. The quality of life after ARDS is significantly reduced with 35% unable to return to work 24 months after hospital discharge [6,7]. ARDS has no primary pharmacological treatments proven to improve outcome other than supportive care with a lung-protective ventilator strategy [8].

Laboratory studies over the last 20 years suggest a potential therapeutic role for β_2 _agonists in ARDS [9]. In brief, evidence suggests these agents reduce alveolar inflammation [10,11], improve endothelial/epithelial barrier function [12,13], accelerate alveolar fluid clearance [14] and enhance epithelial repair [13,15]. A single centre phase 2 trial (Beta Agonist Lung Injury TrIal-1; BALTI-1) investigated the efficacy of intravenous salbutamol on *in-vivo *fluid clearance through serial measurement of extra-vascular lung water in 40 patients with ARDS [16]. The study demonstrated that a sustained intravenous infusion of salbutamol (15 μg kg ideal body weight^-1 ^hr^-1^) over 7 days significantly reduced lung water (day 7 lung water mean (SD), 9.2 (6) vs 13.2 (3) ml kg^-1^, P = 0.038) and plateau airway pressures (23.9(3.8) vs 29.5(7.2) cm H_2_O, P = 0.049). This study provided proof of concept that treatment with intravenous beta agonists may influence alveolar fluid clearance. The study lacked sufficient power to measure the effect on other clinical and cost effectiveness outcomes. The BALTI-2 trial was conceived to test the hypothesis that sustained treatment with intravenous beta agonists in ARDS would improve 28 day mortality and other clinical and cost effectiveness outcomes.

## Methods/Design

### Trial Summary

BALTI-2 is a multicentre, pragmatic, randomised, double-blind, placebo-controlled clinical trial. Patients fulfilling the American-European Consensus Conference Definition of ARDS will be randomised in a 1:1 ratio to receive an IV infusion either of salbutamol (15 μg kg ideal body weight^-1 ^hr^-1^) or placebo (0.9% sodium chloride solution), for a maximum of seven days. Allocation to randomised groups will use minimisation to ensure balance with respect to hospital of recruitment, age group (<64, 65-84, >85 years) and PaO_2_/FiO_2 _ratio (≤6.7, 6.8- 13.2, ≥13.3 kPa). The trial will be fully blinded and all drugs will be packaged identically, so that patients, clinicians or investigators will not know which patients are in each arm. Data will be recorded by participating ICUs until hospital discharge, and all surviving patients will be followed up by post at six and twelve months post randomisation. The primary outcome is mortality at 28 days after randomisation; secondary outcomes are mortality in ICU, mortality in hospital, number of ventilator-free days, number of non-pulmonary organ failure-free days, mortality at twelve months post-randomisation, quality of life at six and twelve months, length of stay in ICU, length of stay in hospital, adverse effects (e.g. tachycardia, arrhythmia, lactic acidosis). 1,334 patients will be recruited from about fifty ICUs in the UK, and an economic evaluation will be conducted alongside the trial.

### Approvals

The trial is approved by Oxfordshire REC "A" (06/Q1604/123) and MHRA CTA number 24698/0004/001 and EudraCT Number: 2006-002647-86. The trial is registered on the International Standard Randomised Controlled Trial Registry (ISRCTN38366450). The trial is co-sponsored by the University of Warwick and Heart of England NHS Foundation Trust (http://www.heartofengland.nhs.uk/ ). The trial is being coordinated by the Warwick Clinical Trials Unit (http://www.warwick.ac.uk/go/ctu). The trial is funded by the Medical Research Council (MRC) and will be conducted in accordance with Good Clinical Practice Guidelines, applicable UK Clinical Trials Regulations and the Standard Operating Procedures of the Warwick Clinical Trials Unit. The trial will be reported in line with the Consolidated Standards of Reporting Trials (CONSORT) 2010 guidelines [17].

### Outcome measures

#### Efficacy

The primary outcome of the study is all cause mortality 28 days after randomisation. Secondary outcomes are mortality before (first) discharge from ICU; mortality before (first) discharge from hospital; number of ventilator-free days; number of non-pulmonary organ failure-free days; duration of ICU and hospital stay; health related quality of life and mortality at twelve months post randomisation.

Ventilator free days were defined in accordance with the ARDSnet criteria [18] as the number of calendar days after initiating unassisted breathing to day 28 after randomisation, assuming a patient survives for at least 48 consecutive hours after initiating unassisted breathing. Un-assisted breathing is defined as one of at least 48 consecutive hours of (1) being extubated with face mask, nasal prong oxygen, or room air (2) T-tube breathing (3)Tracheostomy mask breathing, CPAP = 5 cm H_2_0 without pressure support of intermittent mandatory ventilation assistance.

Non-pulmonary organ failure-free days are defined as the number of days in the first 28 days after randomization that the patient has none of: respiratory support, cardiovascular support, renal support, or neurological support. Organ failure was defined according to Critical Care Minimum Dataset definitions [19].

#### Safety

The frequency with which the following events occur will be reported (1) tachycardia sufficient to stop treatment with trial drug (2) new arrhythmia sufficient to stop treatment with trial drug (3) other side effects sufficient to stop treatment with trial drug (4) serious adverse events and suspected unexpected serious adverse reactions.

#### Others

Health related quality of life will be measured using EQ-5D and SF-12 at six and twelve months after randomisation. Resource use including length of ICU and hospital stay; health service contacts up to twelve months after randomisation; out of pocket expenditure and time away from work.

### Eligibility Criteria

Ventilated patients will be screened daily for the development of ARDS. Adult (age > 16) patients are eligible to be included if they are intubated and ventilated within 72 hours of onset of ARDS. ARDS is defined in accordance with the American-European consensus conference definition of acute onset of severe hypoxaemic respiratory failure (PaO_2_/FiO_2 _ratio ≤ 26.7 kPa) with bilateral infiltrates on the chest radiograph in the absence of clinical evidence of left atrial hypertension. Patients will be excluded if they are known to be pregnant; are receiving current treatment with intravenous β_2_-agonists or have a requirement for on-going regular nebulised/inhaled β_2_-agonists; are being treated with β-adrenergic antagonists ("β-blockers"); treatment withdrawal is imminent; chronic liver disease, defined as Child-Pugh grade C; enrolled in another clinical trial of an investigational medicinal product in the last 28 days;

### Consent

Consent will be sought from the patients themselves if this is possible, but it is recognised that in the majority of cases patients will be unable to give informed consent due to alterations in their level of consciousness caused by illness and therapeutic sedation. In this situation informed consent will be sought from a Personal Legal Representative or Professional Legal representative. Retrospective consent/consent to continue will be sought from surviving patients enrolled through consent from a personal/professional legal representative.

### Randomisation

Randomisation will occur via a central telephone randomization service (University of Aberdeen). Randomisation will be minimised by centre, PaO_2_/FiO_2 _ratio (≤6.7, 6.8 to 13.2, ≥13.3kPa), and age (<64, 65 to 84, ≥85 years) because of the expected differences in mortality among these strata. The randomisation service will allocate a numbered treatment pack to each patient. This pack will contain all drugs for giving a complete course of trial treatment to one patient.

### Drug supply

Drug treatment packs containing sealed and blinded glass ampoules of salbutamol sulphate (GlaxoSmithKline) 5 mg in 5 ml or sodium chloride Injection BP 0.9% w/v (Hameln Pharmaceuticals Ltd) will be supplied by Bilcare GCS (Europe) Limited (Elvicta Business Park, Crickhowell, Powys, UK). All trial drugs will be packaged identically and identified only by number.

Drug infusions will be administered through a dedicated intravenous line at a rate of 0.075 ml (kg ideal body weight)^-1 ^hour^-1 ^(equivalent to 15 μg salbutamol(kg ideal body weight)^-1 ^hour^-1^) according to the patients height (table 1). Trial drug infusions should be started immediately after randomisation. If at the time of attempting to commence the trial drug the patient's heart rate exceeds 140 beats min^-1^, the administration should be delayed until the heart rate is less than 140 beats min^-1 ^for at least 30 minutes. Every attempt should be made to complete the treatment infusion without interruption for a maximum of seven days (i.e. until 168 hours after randomisation).

**Table 1:** Infusion rate according to height (ideal body weight)

Height (cm)	Male IBW (kg)	Infusion Rate (ml hr^-1^)	Female IBW (kg)	Infusion Rate (ml hr^-1^)	Height (cm)	Male (IBW) (kg)	Infusion Rate (ml hr^-1^)	Female IBW (kg)	Infusion Rate (ml hr^-1^)
**146**	44.2	3.3	39.7	3.0	**174**	69.7	5.2	65.2	4.9

**148**	46.0	3.5	41.5	3.1	**176**	71.5	5.4	67.0	5.0

**150**	47.8	3.6	43.3	3.2	**178**	73.3	5.5	68.8	5.2

**152**	49.6	3.7	45.1	3.4	**180**	75.1	5.6	70.6	5.3

**154**	51.5	3.9	47.0	3.5	**182**	76.9	5.8	72.4	5.4

**156**	53.3	4.0	48.8	3.7	**184**	78.8	5.9	74.3	5.6

**158**	55.1	4.1	50.6	3.8	**186**	80.6	6.0	76.1	5.7

**160**	56.9	4.3	52.4	3.9	**188**	82.4	6.2	77.9	5.8

**162**	58.7	4.4	54.2	4.1	**190**	84.2	6.3	79.7	6.0

**164**	60.6	4.5	56.1	4.2	**192**	86.0	6.5	81.5	6.1

**166**	62.4	4.7	57.9	4.3	**194**	87.9	6.6	83.4	6.3

**168**	64.2	4.8	59.7	4.5	**196**	89.7	6.7	85.2	6.4

**170**	66.0	5.0	61.5	4.6	**198**	91.5	6.9	87.0	6.5

**172**	67.8	5.1	63.3	4.7	**200**	93.3	7.0	88.8	6.7

#### Alteration of Infusion Rate

**Figure 1:**
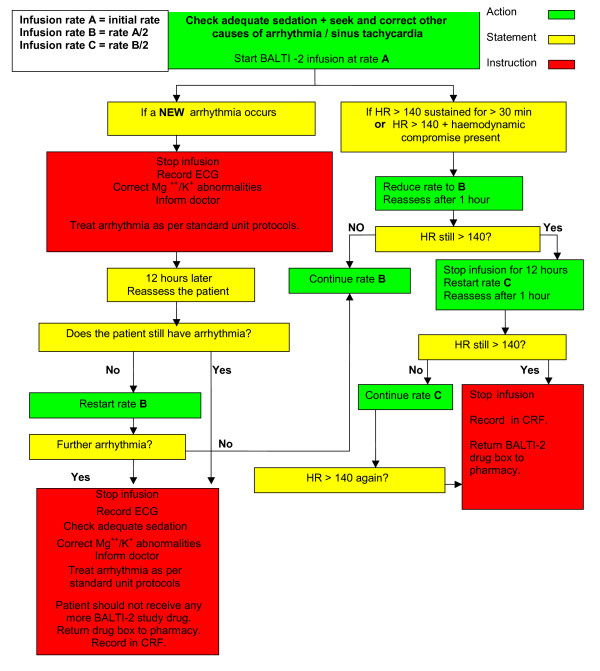
Tachycardiac/arrhythmia management protocol

Sinus tachycardia or arrhythmias are known side effects of intravenous salbutamol administration. If a patient receiving a trial drug infusion is noted to have tachycardia (heart rate > 140 beats min^-1^) or any new arrhythmia occurs, the dose rate of drug will be adjusted according to the flow diagram (figure 1). Dose adjustments for renal or hepatic failure will be driven by the cardiovascular response to the infusion rather than on the degree of renal or hepatic impairment. Standard anti-arrhythmic therapy will be given if indicated in addition to alteration of infusion rate.

#### Infusion Termination Criteria

Termination of the infusion is defined as discontinuation of the trial drug infusion without intention to restart the infusion at a later time. Trial drug infusion will be terminated in the following circumstances: death; heart rate >140 beats min^-1 ^despite two adjustments in infusion rate; new arrhythmias despite adjustment in infusion rate; development of a significant lactic acidosis, which in the opinion of the treating clinician is attributable to infusion of the trial drug; 24 hours after discontinuation of mechanical ventilation (of any sort); discharge from ICU; discontinuation of active treatment; request to withdraw from the personal or professional legal representative; decision by the attending clinician that the infusion should be discontinued on safety grounds; 7 days (168 hours) after randomization.

### Clinical Management of Patients in the Trial

Patients involved in the BALTI 2 trial will be managed according to best practice established locally on each unit. Particular care to monitor electrolytes (K^+^, Mg^++^) and glucose is required, with electrolyte supplementation/insulin administered as clinically indicated. The only specific trial requirement is that patients are not routinely administered nebulised beta agonists or other intravenous beta agonists such as isoprenaline. The uncontrolled use of nebulised bronchodilators in the control group will limit the ability of the trial to detect a significant difference in outcomes and the use in the treatment group exposes the patients to a risk of toxicity. There is no definitive evidence at the current time that routine nebulisation of bronchodilators improves outcomes in patients with acute lung injury. In the event of acute bronchospasm, where the clinician feels that a nebulised bronchodilator is required, nebulised ipratropium bromide may be given. If nebulised ipratropium is insufficient to treat the bronchospasm, then salbutamol may be given as a rescue therapy. This will be recorded on the relevant case report form.

There are no specific guidelines for ventilatory management. Clinicians will be encouraged to use a low tidal volume strategy of ventilation based on ideal body weight. Rescue therapies such as high frequency oscillatory ventilation, nitric oxide and extracorporeal membrane oxygenation can be used according to local policy.

### Post Infusion Follow-up

Any patients who remain in the Intensive Care Unit or High Dependency Unit for more that seven days post randomisation (the end of the expected drug infusion period), will continue to be monitored on daily basis until discharged to a ward. The date and place of hospital discharge will be obtained from hospital records.

All patients discharged from hospital will be followed-up six and twelve months after randomisation by postal questionnaire. The questionnaire will collect data on disability and health-related quality of life, using the EQ-5D and SF-12 questionnaires.

### Adverse Event Management

BALTI-2 is recruiting a population that is already in a life-threatening situation, it is therefore expected that many of the participants will experience serious adverse events. Events that are expected in this population and those that are collected as outcomes (e.g. death, organ failure) of the trial will not be reported as SAEs. Other SAEs or SUSARs that occur between trial entry and 30 days after the end of the trial drug infusion will be reported by faxing a serious adverse event log to the trial co-coordinating centre.

### End of Trial

The trial will end when 1334 patients have been recruited and completed twelve month follow-up. The trial will be stopped prematurely if: mandated by the Ethics Committee or the Medicines and Healthcare products Regulatory Agency (MHRA); following recommendations from the Data Monitoring and Ethics Committee (DMEC); funding for the trial ceases.

### Sample size

Published estimates of the mortality rate among ARDS patients range from about 34% to 60%. Two cohort studies that included UK data estimated that hospital mortality was 53.9% [3] (95% CI 49.0, 58.7%) and 60.9% [4] (95% CI 55.9, 65.9%). However, it is likely that mortality has declined since these studies were conducted (1999) because of the introduction of protective ventilation strategies after the publication of a large RCT in 2000. From unpublished ICNARC data for 2005 [5], the hospital mortality among 37,726 patients with ARDS in the UK was 41.2%. The primary outcome for BALTI-2 is 28-day mortality, which is likely to be similar to or slightly higher than hospital mortality because most deaths will occur in ICU within a short period after randomisation, and most patients leave hospital before 28 days. In BALTI-1 the placebo group 28-day mortality rate was 67% (95% CI 0.45, 0.83). A reasonable conservative estimate of the 28-day mortality to be expected in BALTI-2 is 40-50%.

Losses to follow-up for the primary outcome are expected to be very low; in the recently-completed PAC-Man trial 2.4% of recruited patients were lost (mainly because of withdrawal of consent) between randomisation and hospital discharge. We have therefore conservatively assumed a 3% loss of patients for the primary outcome. Table 2 shows the sample sizes necessary for 80% and 90% power to detect a real risk ratio of 0.80 between the salbutamol and placebo arms, using a significance level of 0.05.

**Table 2:** Required sample sizes for 80 and 90% power, RR 0.80, 3% losses

Placebo mortality	Salbutamol mortality	80% power	90% power
40%	32%	1164	1558

42%	33.6%	1076	1440

**44%**	**35.2%**	**998**	**1334**

46%	36.8%	926	1238

48%	38.4%	860	1148

50%	40%	798	1068

We will adopt a target sample size of 1334, which will give 90% power to detect a risk ratio of 0.8 if the placebo group mortality rate is 44%, over 85% power if it is 40%, and more than 90% if it exceeds 44%. The 28-day mortality in the placebo group will be monitored (via the DMEC), to ascertain whether the assumptions made in the sample size calculations are correct. If not, the DMEC will advise on modification to the sample size.

### Statistical Analysis

#### Primary outcome

Mortality at 28 days post-randomisation will be compared between the groups by the risk ratio and 95% confidence interval. Time to death will also be analysed, using survival analysis methods. The groups will be compared using the hazard ratio and its 95% confidence interval from a Cox-proportional hazards model, and a Kaplan-Meier curve will be used for illustration. The proportional hazard assumption across treatment arms will be checked graphically using a log-cumulative hazard plot. This analysis will be repeated when the long term data are analysed, which will include survival to 12 months for all participants. Dichotomous outcomes (death in ICU, death in hospital, tachycardia, arrhythmia and other side effects) will be compared using risk ratios and 95% confidence intervals. For continuous outcomes (duration of ICU and hospital stay, ventilator-free days and organ failure-free days), mean differences and 95% confidence interval will be presented. Time to event outcomes (length of hospital and ICU stay) will be analysed by survival methods and compared using a hazard ratio and 95% confidence interval. The SF-12 physical and mental component scores will be calculated according to the standard methods [1], and compared between the groups by the mean difference and 95% confidence interval. The EQ-5D will be scored according to the UK valuation model [2,3], and presented as the difference in means between the groups with 95% confidence interval.

#### Subgroup analyses

Four subgroup analyses are planned to analyse whether the treatment effect is modified by age, severity of hypoxaemia before randomisation, aetiology of ARDS, or APACHE II score. Subgroup analyses will be conducted for the primary outcome only. It is proposed to replace the APACHE II score with the APACHE II mortality risk, which is calculated from the APACHE II score but also takes into account the underlying condition. APACHE II scores do not correlate well with mortality risk as similar scores may occur in patients with different conditions who have different risks of mortality. This means that APACHE II score is unlikely to be predictive of outcome or of treatment effect. The mortality risk incorporates the underlying condition and is a better measure of a patient's "sickness". It is therefore more plausible that mortality risk could have a treatment-modifying effect, and it is preferable to explore this variable's relationship to treatment effect. However, APACHE II score will be retained in the table of baseline characteristics as a descriptor of the population recruited to the trial. For aetiology of ARDS, the ratio of risk ratios in the direct and indirect aetiology subgroups will be calculated, with its 95% confidence interval [6]. We will not attempt any analysis of subtypes of direct and indirect aetiologies because the number of patients available is too small and there is a risk of misleading results. The other three subgroup-classifying variables are continuous. Although the BALTI-2 protocol specified categorisations for these variables, it is proposed to adopt a different approach because there are problems with categorising continuous variables. First, there is no clear biological rationale behind any cut-points, and attempting to use data-driven procedures to derive "optimal" cut-points is liable to be seriously misleading. Second, there is good evidence that any form of categorisation of continuous variables is potentially misleading, and better methods are available [7,8]. For these reasons, the potential treatment-modifying factors will not be categorised. Instead, we will use a regression approach to model the interaction between the continuous baseline variables and outcomes in each group. Categorisations of age, severity of hypoxaemia and APACHE II score will be retained in the table of participants' characteristics, to facilitate comparison of the randomised groups. If there appear to be baseline imbalances that could affect the comparison between the groups, exploratory analyses will be conducted, adjusting for these.

### Economic Evaluation

Two economic analyses will be undertaken to calculate the expected incremental cost effectiveness of IV salbutamol compared to standard care in the treatment of patients with ARDS, admitted to ICUs in the UK.

A within-trial cost effectiveness analysis comparing the costs and outcomes of patients in each arm of the trial at 12 months. The perspective for this analysis will be that of the NHS and Social Services. The primary outcome for this analysis will be the Quality Adjusted Life Years (QALY's). Utilities will be measured using the EQ-5D at 6 and 12 months follow-up. Within ICU resource use will be identified through a detailed costing study undertaken at a sample of ICUs recruiting to the trial. Use of other hospital services will be abstracted from the trial CRFs. Use of primary, community and social care services will be recorded via a patient diary completed at six and 12 months follow-up. Particular effort will be made to identify place of residence at 12 months follow-up and whether this is funded by health, social services or privately. Out of pocket expenditure and time away from work data will also be collected using the same patient diary. Unit costs will be obtained from national sources such as the NHS reference costs and the PSSRU Unit Costs of Health and Social Care (http://www.pssru.ac.uk/). Where national costs are not available, unit costs will be identified in consultation with finance departments of trusts recruiting to the trial. Parameter uncertainty will be addressed using probabilistic sensitivity analysis. Outputs from the analysis will include the expected incremental cost effectiveness ratio (ICER), a scatterplot on the cost effectiveness plane, cost effectiveness acceptability curve and incremental net benefit assuming lambda = £20,000 per QALY.

As there is potential for a difference in mortality between the groups, a lifetime horizon is required to fully capture the cost and benefits of IV salbutamol compared to usual care. Therefore, we will construct a cost effectiveness model with a lifetime time horizon. This will model the expected long term difference in QALY's lived and health and social care resource utilised by two hypothetical cohorts of patients with ARDS; one treated with IV salbutamol the other not. The age distribution of these cohorts will reflect the age profile of ARDS patients actually seen in UK ICUs. Life expectancy post hospital discharge will be modelled using national age specific life expectancy data adjusted to reflect published evidence on the reduced life expectancy of ICU 'survivors'. Long- term quality of life will be estimated using published age-specific utility data adjusted to reflect any published evidence of a divergence in health related quality of life in ICU 'survivors'. In the absence of evidence to the contrary, the model will assume that the treatment modality does not impact upon the long terms non-ARDS-related health care costs. Costs and outcomes will be discounted in line with best practice recommendations at the time of the analysis. Parameter uncertainty will be addressed using probabilistic sensitivity analysis. Outputs from the analysis will include the expected ICER, a scatterplot on the cost effectiveness plane, cost effectiveness acceptability curve and incremental net benefit assuming lambda = £20,000 per QALY.

### Trial oversight

The day to day management of the trial will be undertaken by a trial management group comprising chief investigator, co-investigators and the trial team. Trial oversight will be provided by a Trial Steering Committee comprised of investigators and independent members. The committee will advise upon major decisions such as a need to change the protocol for any reason; monitoring and supervising the progress of the trial; Reviewing relevant information from other sources; Considering recommendations from the Data monitoring and ethics committee (DMEC); Informing and advising on all aspects of the trial.

An independent DMEC will be appointed. They will be responsible for monitoring data accumulating from the trial. Interim analyses are anticipated to take place every 12 months during the period of recruitment, or more frequently if requested by the DMEC. The DMEC will advise the Chairman of the Steering Committee if, in their view, the randomised comparisons have provided both (i) 'proof beyond reasonable doubt' that for all, or some, the treatment is clearly indicated or clearly contra-indicated and (ii) evidence that might reasonably be expected to materially influence future patient management. Following a report from the DMEC, the Steering Committee will decide what actions, if any, are required. Unless the DMEC request cessation of the trial the Steering Committee and the collaborators will remain ignorant of the interim results.

## List of Abbreviations

ALI: Acute lung injury; APACHE II: Acute physiology and chronic health evaluation II; ARDS: Acute respiratory distress syndrome; CI: Confidence interval; CONSORT: Consolidated Standards of Reporting Trials; CRF: Case report form; CTA: Clinical Trials Authorisation; CTU: Clinical Trials Unit; CXR: Chest X-ray; DMEC: Data Monitoring and Ethics Committee; EQ-5D: EuroQol 5 dimension questionnaire; GCP: Good Clinical Practice; ICNARC: Intensive Care National Audit and Research Centre; ICU: Intensive Care Unit; ISRCTN: International Standardised Randomised Controlled Trial Number; IV: Intravenous; MHRA: Medicines and Healthcare products Regulatory Agency; NHS: National Health Service; QALY: Quality adjusted life years; SAE: Serious adverse event; SF-12: Short form 12 questionnaire; SUSAR: Suspected unexpected serious adverse reaction; VFD: Ventilator free days.

## Competing interests

In the last 5 years GDP and FG received a research grant from the manufacturers of salbutamol (GlaxoSmithKline) to investigate the effects of salmeterol on alveolar inflammation in ARDS. GDP has received lecture fees and reimbursement of expenses from GlaxoSmithKline.

## Authors' contributions

GDP and FG conceived the study. All authors made a substantial contribution to the protocol development. All authors have approved this manuscript.
